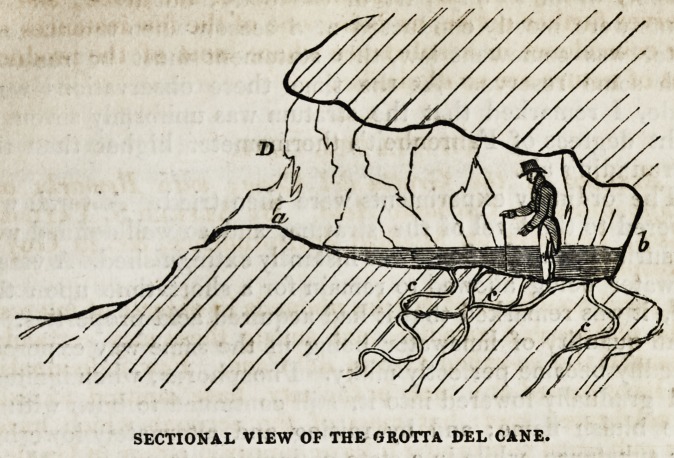# An Account of the Grotta Del Cane; with Remarks on Suffocation by Carbonic Acid

**Published:** 1832-10

**Authors:** Alfred S. Taylor

**Affiliations:** Lecturer on Medical Jurisprudence, &c. in Guy's Hospital.


### GROTTA DEL CANE.

*An Account of the Grotta del Cane; with Remarks on Suffocation by Carbonic Acid.* By Alfred S. Taylor, Esq., Lecturer on Medical Jurisprudence, &c. in Guy's Hospital.

The neighbourhood of Naples abounds in objects of interest to the philosophic traveller, and among these may certainly be ranked the so-called Grotta del Cane. Few individuals, who have passed any time in the city, have omitted to visit this celebrated spot; but most of the accounts which have been published concerning it have appeared either in Guide-books or Itineraries, and have, therefore, been altogether destitute of that precision which the chemist or philosopher naturally looks for in the description of such subjects.*

During a residence of some months in this part of Italy, I took the opportunity of paying several visits to the Grotta del Cane, in order to satisfy myself of the truth of the accounts which I had seen published. The origin of the name has long been familiar to the tyro in chemistry. It was probably in this spot that the spontaneous production of carbonic acid from the earth was first noticed; and the experiments being made for the most part on dogs, soon gave to the locality that name which it has since retained, and by which it is universally known. The grotto itself is simply an excavation in the side of a hill, formed of primitive tufa, situated on the border of the Lake of Agnana, and was probably made at some remote period for the purpose of extracting *puzzuolana*. There is every reason to believe that it was not excavated for the purposes for which it is now so celebrated, although some have stated the contrary. The cave, of which the accompanying diagram presents a longitudinal sectional view, is, as will be perceived, of an irregular form, being about twelve feet in length, four and a half in breadth, and about five feet in height. It is lower at the back part than in front, so that an individual of mean stature is unable to stand erect within it.

* In order to shew the inaccuracy of the accounts published concerning the Grotta del Cane, I may mention that a classic traveller in Italy has recently, in his Travels, described the "grotto" to be a cave emitting "powerful *sulphureous exhalations;*" and he further states, that he himself was comparatively choked and stifled by the "*sulphuric* fumes!"

SECTIONAL VIEW OF THE GROTTA DEL CANE.

The sides are formed of the crumbling volcanic tufa, which has somewhat the appearance of clay; but it is not discoloured by the carbonic acid, as has been erroneously asserted by Dr. Mead. On the floor of the grotto, which is irregularly inclined downwards from the entrance *D*, exists the stratum of gas, the level of which is indicated in the diagram by the lines drawn from *a* to *b*. In consequence of this inclination of the floor, it will be seen that a sort of reservoir or basin is formed, in which the gas collects. From this cause the stratum is unequal in depth: in the fore part of the grotto it rises only to a few inches above the surface of the soil, while in the back part it reaches to nearly fourteen inches; but it is never observed to extend above the level *a*, *b*: for if, at any season of the year, it be poured out in greater abundance than usual, this additional quantity, in consequence of the great density of the gas, readily flows over at *a*, and thus escapes from the grotto.

The properties of this stratum of gas are somewhat peculiar. It may be observed to issue, under ordinary circumstances, from crevices or fissures in the soil, especially towards the deeper part of the grotto; and it may be readily recognized by the quantity of vapour with which it is combined, which, by its condensation, gives to the whole the appearance of steam. The hygrometer of Saussure, when held for a short time within it, fell to the zero of humidity; and cold bodies, suddenly introduced, became rapidly covered with the condensed vapour. Its temperature is so much higher than that of the surrounding atmosphere, that it feels sensibly warm to any part of the body; but as my experiments were mostly conducted at a season when the thermometer was somewhat low, this statement must be received with some reserve. At the time these observations were made, I remarked that the stratum was uniformly seven or eight degrees of Fahrenheit's thermometer higher than the surrounding air.

The ordinary experiments were then tried. A torch was lowered to the level of the stratum, and so well defined was its surface that the flame was instantly extinguished. A vessel of water being allowed to remain for a short time upon the soil, it was remarked that it had acquired acid properties. A small quantity of limewater being in the same way exposed, speedily became perfectly milky. Phosphorus, when ignited, and gradually lowered into it, still continued to burn with a pale bluish flame; and by raising and alternately lowering this substance, while in a state of ignition, it was possible to define clearly the exact height of the stratum. A dog was now introduced, and forcibly retained below the level of the gas, near the entrance of the grotto. The animal made violent attempts to escape: its respiration became at first difficult; the tongue became swollen, and finally protruded from the mouth; the nostrils were covered with a frothy mucus; the eyes seemed to project from the orbits, and assumed a peculiarly bright lustre. The faculties of sensation and voluntary motion now ceased: the limbs became convulsed and drawn backwards, the chest heaved spasmodically at intervals, and the animal seemed, indeed, about to expire. These phenomena, following each other with rapidity, did not occupy more than two minutes of time: the dog was then removed from the grotto, and placed in a free current of air. The powers of life now slowly returned; but the animal seemed to be labouring under a kind of stupor, during which he was unable to support his body, and repeatedly fell to the ground in his attempts to move. There was no individual present willing to undergo the experiment, to the same extent, on his own person: but the well-known impression produced by carbonic acid on the Scheiderian membrane was perceived when the face was brought to the level of the gas.

When a portion of this elastic fluid was submitted to chemical examination, it was found not to consist of pure carbonic acid, as has been erroneously stated by numerous authors; for it was proved to contain 0.06 of atmospheric air. Prof. Breisluk affirms that it contains also a notable quantity of pure nitrogen: this, however, could not be detected; nor did the specimen which I examined contain any indications of the presence of sulphuretted hydrogen.

The true origin of this quantity of carbonic acid is a point involved in some mystery. Those who have paid close attention to the geological character of the district in which Naples is situated, agree in considering that, in the immediate neighbourhood, volcanic action, although quiescent on the surface, is still going on beneath. The primitive tufa, as it has been termed by Professor Tenore, in which the Grotta del Cane is situated, is interposed between a series of extinct and half-extinct volcanic foci. The city of Naples itself occupies a part of the bed of the former, while the town of Pozzuoli is immediately surrounded by the latter. Now there are few spots in the immediate vicinity of Pozzuoli, where, by digging to any depth, we may not obtain either muriatic acid vapour, sulphuretted hydrogen, or carbonic acid. The Grotta del Cane may be looked upon as an excavation of this nature upon the borders of a lake, which many have supposed, from its very regular form, and from the manner in which it is surrounded by a well-defined crest or ridge of volcanic materials, to be nothing less than the crater of an extinct volcano. Within a short distance, and in a line with the Grotta del Cane, sulphuretted hydrogen gas issues out of the ground in vast quantities, and has given rise to the establishment of the *Stufe*, or Baths of San Germano: and immediately adjoining is the crater of the Solfatara, the existence of which as an active volcano, at no very remote period of history, appears now to be universally admitted. We may, then, readily understand that these peculiar gases, which are constantly forming at a greater or less depth below the surface of the soil, are among the last results of expiring volcanic action. When formed, they find their way readily through fissures in the strata, as represented at *c, c*, and afterwards collect in cavities on the surface, in greater or less quantity, according to their particular form or inclination. Thus the carbonic acid, generated at an unknown depth, rises to the surface of the soil in the grotto, and becomes collected in consequence of its peculiar form. If, therefore, the floor of the grotto were made perfectly level, or but slightly inclined from the entrance, there would be an end to all those phenomena for which it is now so celebrated.*

With this brief account of a spot which has been thought worthy of notice by almost every chemical writer, I shall pro-

* The proprietor is enabled to maintain himself and family, and a necessary number of dogs, by the profits resulting from the exhibition of the grotto.

ceed to offer a few remarks upon some of the phenomena attendant on suffocation by carbonic acid in man.

Where this gas has been inspired in a state of great purity, death is an almost instantaneous result. It is seldom that any sound is uttered prior to dissolution, or that the being gives any indication of pain or suffering. According to the experiments of Sir HUMPHREY DAVY, the gas does not, under these circumstances, penetrate into the pulmonary organs, the glottis closing spasmodically, and obstructing its entrance; but this experimentalist perceived a strong and pungent sensation in the mouth and fauces, while the gas had a distinctly acid taste. When diluted with air, the symptoms which it produces are those of narcotism, and the patient commonly dies apoplectic.* The incipient symptoms of poisoning by carbonic acid are not very often witnessed; but, from cases which have been published by M. COLLARD DE MARTIGNY and others, in the Archives Générales de Médecine, it would appear that giddiness, vertigo, ringing in the ears, with indistinctness of vision, commonly precede the state of insensibility in which those who have respired it have been found. Convulsions have not unfrequently been observed, and in those cases in which recovery has taken place there has often remained a partial paralysis of the muscles of the face or limbs, with stupor and headach.

The post-mortem appearances exhibited in the bodies of those who have been poisoned by carbonic acid, are not of a very definite character. The surface has been usually found more or less livid, especially the face, which is described as being composed and tranquil. The animal heat is long retained, and consequently the cadaverous rigidity of the muscular system is a much longer time than usual in developing itself. The right side of the heart, and the great vessels connected with it, have been found gorged with blood. The vessels of the lungs and of the brain have also been found in the same state by various observers; but in the few experiments which I have made on animals, I have rarely witnessed that engorgement of the cerebral vessels which has been described by PORTAL and MERTZDORFF. Where the lungs were excessively distended, the vessels of the brain contained but a small quantity of blood; and this I had occasion to observe more particularly in those cases in which the acid had

* These effects were observed to follow where three parts of the gas were diluted with seven of common air. It has been suggested by Dr. PARIS, that a compound of this nature might be advantageously administered in certain diseases where sedatives are indicated; but I am not aware that this suggestion has ever been followed in practice.

proved fatal when respired in a diluted state. The appearances, it will be seen, do not differ from those of asphyxia generally; and hence, for the detection of the cause, we must rely upon general or circumstantial evidence.

There has been great variety of sentiment among experimentalists as to the precise manner in which this gas produces a deleterious action on the economy. By some it has been regarded as a directly poisonous agent; while Nysten, who is followed by Orfila, considers that the results of his experiments have established the contrary. This physiologist, who experimented largely on the gases, with a view to arrive at some precise data, in order to determine their mode of action, came to the conclusion that carbonic acid, like nitrogen and hydrogen, destroyed life merely by its negative influence on the system.

In order to determine the relatively poisonous properties of gases, Nysten adopted the plan of injecting them, in certain quantities, into the blood-vessels. In this way he was led to entertain the opinion that, while some exerted a specific influence, giving rise to peculiar symptoms, which were at the same time the rapid forerunners of death, others seemed to act more slowly, and to possess no essentially deleterious properties. Among the latter he placed carbonic acid, and his conclusions with regard to the influence of this gas on the animal economy are, 1st, that it may be injected in comparatively large quantities into the venous system, without interfering with the circulation; 2d, that it exerts no primary action on the brain, but, when injected in a greater quantity than can be held in solution by the circulating fluids, it gives rise to a violent distention of the texture of this organ, and causes death by apoplexy; 3dly, that, when injected under proper precautions, it produces merely a general debility of the muscular system, which passes off after a few days. In the performance of these experiments, unless the gas be thrown in with care, death may result from the mechanical violence produced by the forcible introduction of an elastic fluid into the blood-vessels; but this effect must be contradistinguished from those effects which result from the specific properties of the gas, since in this way the life of an animal may be as surely and as rapidly destroyed through the injection of pure atmospheric air, as through the injection of any other elastic compound, however deleterious.

The conclusions of Nysten were for a long time regarded as correct, and physiologists readily embraced the opinion he had formed, namely, that carbonic acid was not, *per se*, a poisonous agent. More recent observations have, however, led to more perfect views on the subject; and, although many still adhere to the old opinion, yet the majority of those who have deeply reflected on the subject seem now to agree that carbonic acid has a directly poisonous action on the economy. Nysten himself admitted that, among those gases which he had classed as negatively destructive, there were some which, when introduced into the lungs, appeared to act in a different manner to others. Thus the carbonic oxide was found to destroy life more quickly than nitrogen or the nitrous oxide; while the proto-phosphuretted hydrogen and carbonic acid act with greater rapidity than the carbonic oxide. Now, it is impossible to reconcile these differences with the supposition that the action of the gases above mentioned is merely negative; and hence we must be prepared to admit that they possess certain properties, which more or less distinguish them from each other. If, for instance, it be affirmed that the action of carbonic acid on the system differs not from that of nitrogen, it ought necessarily to follow that these gases may mutually replace each other; but this, by experiment, is not found to be the case. M. Collard de Martigny has ascertained that an atmosphere formed of carbonic acid and oxygen cannot be respired, even for a very short period of time, without producing fatal effects; and from this it is reasonable to conclude that its operation on the economy must be of a positively deleterious nature. This conclusion is still further strengthened by other experiments, undertaken by the same physiologist. Having enclosed animals in an atmosphere of carbonic acid, he allowed them to respire freely the pure atmospheric air; but still he found that the usual symptoms produced by the action of this gas on the economy manifested themselves, although it had not entered into the respiratory organs, and therefore could not have acted in the manner assumed by those who maintain an opposite opinion. If it be a fact that carbonic acid is capable of exerting its peculiar influence through any texture of the body, there can be no longer a doubt as to which side of the question our judgment should incline. The intoxicating power of certain fluids impregnated with this gas, as well as the dizziness, vertigo, and other symptoms of cerebral disturbance, which the long-continued use of them induces, have been long known; but it has been only recently suspected that the carbonic acid, by its action on the mucous membrane of the stomach, may have a considerable share in the production of these symptoms.

Without dwelling upon the importance of these observations, it will be sufficient to conclude this paper by a notice of the very ingenious experiments recently performed by Professor

Rolando, of Turin; for this, even if all other proofs were wanting, is sufficient to set the question at rest.* This distinguished physiologist, having separated the bronchi in a tortoise, succeeded in making it respire carbonic acid by one lung, and atmospheric air by the other: the animal died in the course of a few hours. To this experiment it may be objected that the respiratory process was impeded throughout one half of the pulmonary system; and that, therefore, the animal may have perished, not from any positive action of the carbonic acid, but in consequence of the defective manner in which the process of respiration was necessarily conducted. In order to silence at once all objections on this head, the Professor next secured by a ligature one of the bronchial tubes, having separated it from its fellow; and he found that the effects produced on the animal by the operation were but slight. The inference which he drew from the results of these interesting experiments was precisely that which was deduced from the preceding observations; namely, that carbonic acid has a directly deleterious influence upon the animal functions, in whatever way it may be introduced into the system.

* This experiment is mentioned by Dr. Christison, at page 596 of his admirable Treatise on Poisons.

## Figures and Tables

**Figure f1:**